# Pitfalls of Preoperative Embolization for Meningiomas: A Case Report on Occult Anastomosis Between the Middle and Posterior Meningeal Arteries

**DOI:** 10.7759/cureus.80182

**Published:** 2025-03-06

**Authors:** Hirokuni Hashikata, Gen Futamura, Yoshinori Maki, Hideki Hayashi, Hiroki Toda

**Affiliations:** 1 Department of Neurosurgery, Medical Research Institute Kitano Hospital, Public Interest Incorporated Foundation (PIIF) Tazuke-Kofukai, Osaka, JPN; 2 Department of Neurosurgery, Ayabe Renaiss Hospital, Ayabe, JPN; 3 Department of Neurosurgery, Hikone Chuo Hospital, Hikone, JPN

**Keywords:** ischemic complication, middle meningeal artery, occult anastomosis, posterior meningeal artery, preoperative embolization

## Abstract

Preoperative embolization facilitates meningioma resection by reducing intraoperative bleeding. The middle meningeal artery (MMA) is frequently targeted; however, the presence of occult anastomoses between the MMA and the posterior meningeal artery (PMA) and their potential involvement in ischemic complications remain poorly understood.

A 61-year-old woman with no prior medical history was incidentally found to have a right parietal extra-axial tumor on magnetic resonance imaging (MRI). She was neurologically intact upon admission, but perifocal cerebral edema was observed surrounding the tumor, which measured approximately 33 mm × 27 mm × 22 mm. Given the patient's condition and life expectancy, tumor resection was planned, preceded by preoperative embolization to minimize intraoperative bleeding.

Angiography identified the parieto-occipital branch of the right MMA as the primary feeder, and superselective angiography showed no evidence of anastomoses. Microparticles were injected into the MMA; however, immediately afterward, the patient became comatose and exhibited transient dysarthria. Postembolization angiography revealed retrograde visualization of the vertebrobasilar system through previously undetected MMA-PMA anastomoses. Subsequent MRI confirmed extensive ischemic lesions in the posterior lobes, hippocampus, cerebellum, and thalamus.

The patient later developed right hemiparesis, clumsiness, dysarthria, and memory impairment, necessitating rehabilitation. While motor symptoms resolved within 10 days, memory disturbance persisted but improved with therapy.

This case highlights the risk of ischemic complications due to occult MMA-PMA anastomoses, which may become functionally significant only during embolization, even if undetectable on preprocedural superselective angiography. This dynamic vascular response underscores the need for heightened vigilance in preoperative embolization planning. Clinicians should consider the possibility of latent anastomoses emerging intraoperatively, necessitating careful embolic agent selection and procedural modifications to minimize complications. Recognizing this risk may enhance patient safety and optimize embolization strategies for meningioma treatment.

## Introduction

The middle meningeal artery (MMA) is typically a branch of the external carotid artery (ECA). The MMA primarily arises from the internal maxillary artery (IMA), occasionally originates from the internal carotid artery (ICA), ophthalmic artery (Opth A), or basilar artery (BA) due to embryological variations [1]. It enters the cranial cavity through the foramen spinosum and bifurcates into anterior and posterior divisions [1]. The MMA has several branches: the petrosal branch which supplies the trigeminal ganglion, the superior tympanic artery which vascularizes the facial nerve and tympanic cavity, the cavernous branch which supplies the cavernous sinus, and the medial branch which serves the sphenoid wing and superior orbital fissure [1]. The parieto-occipital and petrosquamosal branches contribute to the occipital dura, transverse sinus, and sigmoid sinus [1].

The MMA anastomoses with the Opth A, ICA, and posterior meningeal artery (PMA), making it clinically significant in meningioma embolization and chronic subdural hematoma treatment [1]. Preoperative endovascular embolization of meningiomas is a widely recognized adjunctive technique that facilitates surgical resection by reducing intraoperative bleeding and shortening operative time [1-4]. The MMA is often the primary vascular supply to meningiomas and is a common target for embolization. Despite the benefits of this procedure, concerns regarding its safety have been raised, particularly regarding the risk of ischemic complications [5-8]. While ischemic complications related to MMA embolization have been reported in case series and retrospective surveys [5,9-16], the potential risks associated with anastomoses between the MMA and the PMA remain underexplored.

Anastomoses between different arterial networks in the cranial vasculature are not uncommon [1]. However, these vascular connections may be small, functionally dormant, or undetectable on conventional angiographic studies [1,4]. Under certain hemodynamic conditions, such as those induced by embolization, previously occult anastomoses may open and allow embolic materials to travel to unintended regions, causing ischemic injury [1,5]. This phenomenon has been reported in relation to various arterial territories, but its implications for MMA-PMA anastomosis remain poorly understood [1,4,5].

In this case report, we describe a rare ischemic complication resulting from an occult anastomosis between the MMA and PMA in a patient undergoing preoperative embolization for a convexity meningioma. Despite performing superselective angiography of the MMA prior to embolization, the presence of this anastomosis was not detected, leading to the retrograde flow of embolic material into the vertebrobasilar system. This case highlights the potential pitfalls of MMA embolization, emphasizing the need for heightened vigilance and refined angiographic techniques to detect and mitigate such risks.

## Case presentation

**Figure 1:**
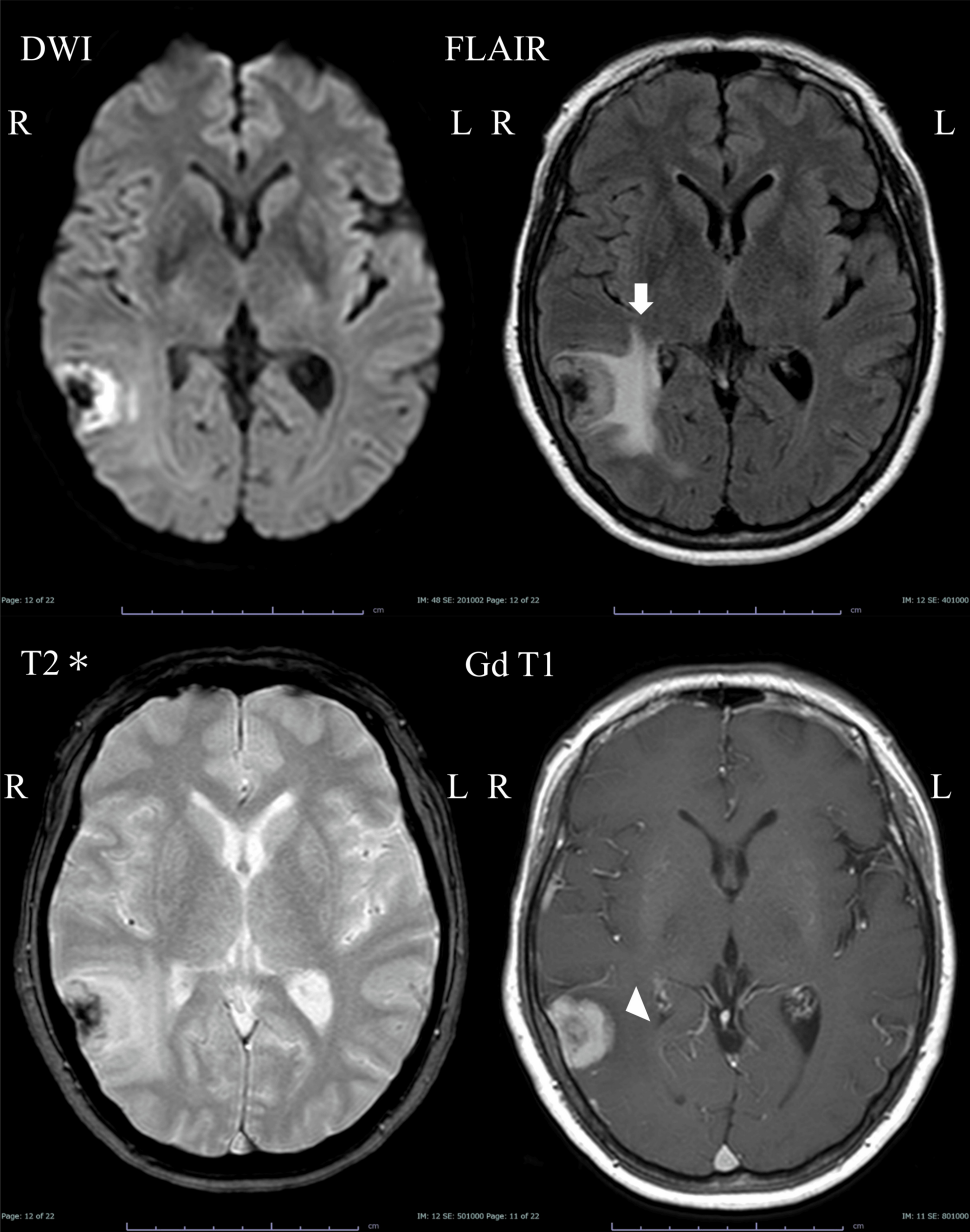
Initial magnetic resonance images. The middle cranial fossa is accompanied by perifocal cerebral edema (white arrow) with an extra-axial mass (white arrowhead). DWI: diffusion-weighted image, FLAIR: fluid-attenuated inversion recovery image, T2*: T2 star-weighted image, Gd T1: gadolinium contrast-enhanced T1-weighted image.

This case report was conducted in accordance with the ethical guidelines and approved by the Institutional Review Board at the Medical Research Institute Kitano Hospital, Public Interest Incorporated Foundation (PIIF) Tazuke-Kofukai. The study adhered to the ethical principles outlined in the Declaration of Helsinki, ensuring that all procedures were performed with respect for the rights, safety, and well-being of the patient. Written informed consent was obtained prior to the treatment. A previously healthy 61-year-old woman was incidentally found to have a right parietal extra-axial tumor on magnetic resonance imaging (MRI). The patient remained neurologically intact on admission. The gadolinium contrast agent used during imaging homogenously enhanced the tumor, measuring approximately 33 mm × 27 mm × 22 mm, with a dural tail sign. The tumor showed calcification near the dura and was accompanied by perifocal cerebral edema (Figure 1).

**Figure 2:**
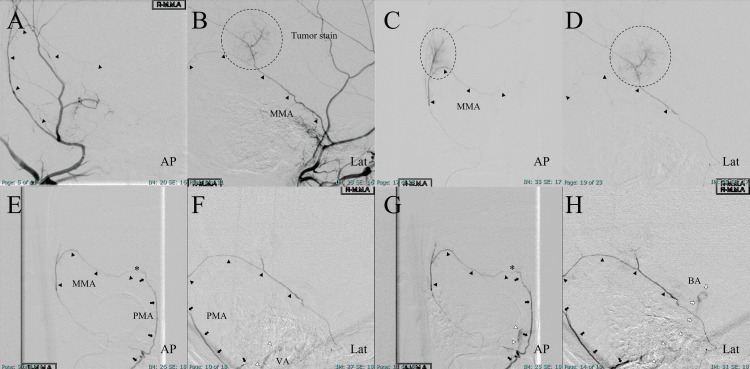
Angiographic findings; embolization of the MMA. Parieto-occipital branch of the MMA-fed tumor (A, B). Superselective angiography before embolization (C, D). After embolization (E, F: early phase; G, H: delayed phase). Black arrowhead: Parieto-occipital branch of the MMA, black arrow: PMA, white arrowhead: VA, white arrow: BA, asterisk: occult anastomosis, broken circle: tumor stain and feeding artery. AP: anteroposterior projection, Lat: lateral projection, MMA: middle meningeal artery, PMA: posterior meningeal artery. VA: vertebral artery, BA: basilar artery.

Based on MRI findings, the preoperative diagnosis was meningioma. A multidisciplinary discussion was held among neurosurgeons and neurointerventional specialists at our institution. Given that the patient was asymptomatic but exhibited perifocal edema, had a life expectancy exceeding 20 years, and maintained independent activities of daily living (ADL), it was deemed appropriate to recommend tumor resection. The patient agreed with this approach, and a treatment plan was established, including tumor resection and preoperative tumor embolization to minimize intraoperative bleeding. Based on this decision, angiography was performed to evaluate the vascular supply of the tumor and determine the embolization strategy. Right external carotid angiography revealed that the parieto-occipital branch of the MMA fed the meningioma (Figures 2A-2B). Conversely, the right internal carotid angiography did not reveal any tumor staining. A 5 Fr long sheath (Radifocus introducer 25 cm; Terumo Corp., Tokyo, Japan) was inserted into the right femoral artery. The right ECA was selected using a coaxial system involving a 5 Fr guiding catheter (Envoy STR 90 cm; Cordis Endovascular, Miami, FL, USA) combined with a catheter (3.6 Fr OK2M 125 cm; KATECS, Osaka, Japan) and guidewire (0.035-inch Radifocus guidewire 150 cm; Terumo Corp., Tokyo, Japan). Heparin was injected intravenously and the activated clotting time was maintained at approximately 250s. A distal access catheter (TACTICS microcatheter; Technocrat Corporation, Aichi, Japan) was guided to the MMA using a micro catheter (Headway Duo; Terumo Corp., Tokyo, Japan) and a micro guidewire (ASAHI CHIKAI 10; Asahi Intecc, Aichi, Japan). Selective MMA angiography confirmed that the parieto-occipital branch of the MMA fed into the meningioma. Thereafter, the microcatheter was guided to the parieto-occipital branch of the MMA. Power-injected superselective angiography of the parieto-occipital branch revealed a tumor stain, with no other arterial anastomoses (Figures 2C-2D). Tris-acryl gelatin microspheres (Embosphere; Merit Medical Systems, South Jordan, Utah, USA) was selected as the embolization material; after a 20-fold dilution (100-300 µm particles) with a non-ionic contrast medium, it was applied to embolize the feeding artery. The diluted Embosphere was injected gradually to confirm whether retrograde flow or erroneous injection of the embolization material would be observed under real-time digital subtraction fluoroscopy (i.e., the “road-mapping” technology). The patient underwent neurological examination at every 4 mL injection of the diluted Embosphere; overall, 20 mL of the diluted Embosphere was injected, resulting in a reduced tumor stain. However, the patient suddenly fell unconscious after the final Embosphere injection and briefly exhibited dysarthric speech before becoming comatose. Right MMA angiography revealed a retrogradely visualized BA via the PMA (Figures 2E-2H).

**Figure 3:**
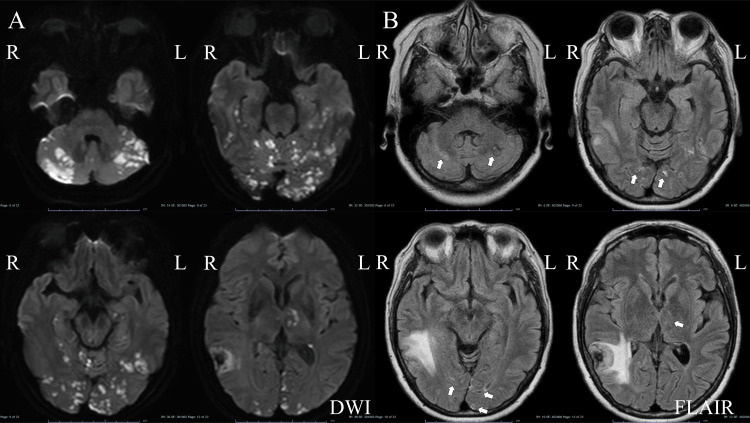
Magnetic resonance images obtained after middle meningeal artery embolization. Diffused ischemic lesions in the posterior cerebral lobes, cerebellar hemispheres, and thalami (A). FLAIR images obtained one year after ischemic complications (white arrow) (B). DWI: diffusion-weighted image, FLAIR: fluid-attenuated inversion recovery image.

Post-embolization MRI revealed diffuse ischemic lesions in the occipital lobes, bilateral hippocampus, left thalamus, and cerebellar hemispheres (Figure 3A). One hour later, the patient became lethargic and developed right hemiparesis, clumsiness, and dysarthria. Although she fully recovered from these conditions 10 days after embolization, the memory disturbance persisted. The patient was transferred to a rehabilitation facility two weeks after embolization. After rehabilitation therapy, her memory disturbance remitted. A follow-up MRI did not reveal any ischemic lesions (Figure 3B).

One year after the embolization, the meningioma was surgically removed. The patient was neurologically intact and experienced no tumor recurrence three years after surgery.

## Discussion

The present case highlights the risk of ischemic complications due to occult anastomosis between the MMA and PMA following preoperative embolization of meningiomas. This anastomosis remained undetected despite superselective angiography of the parieto-occipital branch of the MMA. Embolic materials may retrogradely migrate into the BA through anastomosis between the MMA and PMA. To our knowledge, this has not been previously reported.

In our case, the parieto-occipital branch of the MMA was the main feeder of the meningioma and targeted for embolization. This branch frequently anastomoses with the PMA [1,17,18]. Despite superselective angiography to rule out dangerous anastomoses [19,20], an occult MMA-PMA anastomosis led to ischemic complications. Failure to visualize this connection pre-embolization was critical. A broader angiographic approach, including whole-brain imaging, multiple oblique views, or altered hemodynamic assessments, might have improved detection.

The MMA forms key anastomoses with branches of the ICA, ophthalmic artery (Oph A), ECA, and vertebrobasilar system, which are crucial in embolization, arteriovenous fistulas (AVFs), and vascular pathology. One major connection is with the Oph A, where the anterior MMA links to the recurrent meningeal artery, posing a risk of ocular ischemia. Another significant anastomosis exists between the MMA and the ICA's inferolateral trunk, forming a network around the cavernous sinus. The petrosal branch of the MMA also connects with the ascending pharyngeal, occipital (mastoid branch), and stylomastoid arteries, supplying the jugular foramen and temporal bone.

The PMA, a branch of the vertebral artery (VA), connects with the posterior MMA, supplying the posterior fossa and transverse sinus, necessitating caution in embolization to prevent posterior circulation infarction. These connections emphasize the need for thorough preoperative angiographic evaluation to prevent ischemic complications affecting the brain, cranial nerves, or orbit. Our report suggests that even if anastomoses are not visible on superselective angiography before embolization, they may become evident during the procedure.

Liquid embolic agents such as n-butyl cyanoacrylate (NBCA) and ethylene vinyl alcohol copolymer (Onyx) are increasingly used for their deep penetration into tumor vasculature [14-16]. Unlike particulate embolics such as polyvinyl alcohol (PVA) or tris-acryl gelatin microspheres (TAGM, Embosphere), which occlude proximal vessels, liquid embolics polymerize in situ, ensuring more complete and durable occlusion [3,15], reducing the risk of incomplete devascularization [13].

NBCA minimizes embolic migration through undetected anastomoses by forming a stable polymerized cast, reducing non-target embolization risk, which is crucial in cases with occult MMA-PMA anastomoses [4,16]. Conversely, small PVA particles may inadvertently pass through undetected anastomoses, increasing non-target embolization risk [19].

Liquid embolics offer superior fluoroscopic visibility, aiding real-time monitoring and preventing reflux. NBCA mixed with Lipiodol becomes radiopaque, enhancing intraoperative detection [15], while Onyx allows gradual injection for better control. Particulate agents, being radiolucent, require contrast for tracking, limiting real-time detection of reflux and anastomotic flow [15].

However, NBCA use is technically demanding, requiring precise catheter placement and controlled injection speed to prevent reflux into normal arteries [14]. Onyx may also track along anastomotic pathways, increasing cranial nerve ischemia risk in skull base meningiomas [8].

In our case, where an occult MMA-PMA anastomosis was undetected, NBCA might have reduced ischemic complications by minimizing distal embolization. However, its technical challenges and risks warrant careful consideration. Further studies should assess optimal embolization strategies, balancing safety, efficacy, and feasibility in high-risk anastomotic networks.

A routine six-vessel study (bilateral ICA, ECA, and VA angiography) is essential before embolization to identify anastomoses [14], but occult anastomosis may still be undetected intraoperatively. Particulate embolic materials pose a higher migration risk through unrecognized pathways, complicating cases with subtle collateral flow. While superselective angiography remains standard, some anastomoses may become functionally significant only after embolization alters vascular flow. More dynamic intraoperative assessments, such as repeated contrast injections at different stages, could help prevent ischemic events. A heightened awareness of these risks and a more cautious embolization strategy may reduce complications. Additionally, intraoperative migration of particulate embolic material cannot be directly observed on digital subtraction angiography. The use of tris-acryl gelatin microspheres in our case likely contributed to ischemic complications. If an occult anastomosis is suspected, repetitive superselective angiography or provocation tests should be considered [14].

As for a technical issue in our case, embolic material was injected proximal to the bifurcation of the parieto-occipital branch of the MMA. Vascular tortuosity prevented distal microcatheter placement, so embolization could only be performed from a more proximal position, which may have activated an occult MMA-PMA anastomosis. Akimoto et al. recommended placing microcatheters close to the tumor to minimize this risk [2]. Optimizing microcatheter placement reduces reflux risk into anastomotic pathways but carries potential risks such as vascular laceration or excessive embolic penetration [15]. Further studies should examine embolization site selection and ischemic complications from occult anastomoses.

Several studies have documented ischemic complications following preoperative meningioma embolization, emphasizing meticulous technique and embolic material selection. Iacobucci et al. assessed 64 patients undergoing preoperative embolization using PVA particles and reported a 4.7% minor complication rate [5]. A systematic review by Ilyas et al. found an overall complication rate of 12%, with major complications occurring in 6% [6]. Przybylowski et al. analyzed 28 skull base meningioma cases and found a 4% major complication rate due to peritumoral edema following Onyx embolization [8]. Carli et al. reported a 5.6% complication rate in a series of 201 embolized meningiomas [11]. Becker described delayed hemorrhage, likely due to reperfusion injury and vascular fragility from incomplete devascularization [19]. Akimoto et al. noted that proximal embolization may increase anastomotic recruitment, leading to non-target embolization [2]. Beyond ischemia, cranial nerve deficits have been reported. Jumah et al. described neuropathy from liquid embolic agents in skull base meningiomas due to inadvertent embolization of cranial nerve-supplying vessels [7]. Singla et al. found that meningiomas with significant pial supply had a higher risk of stroke and hemorrhage, necessitating careful angiographic assessment [16].

These studies highlight the multifactorial risks of preoperative embolization, including ischemia, cranial nerve injury, and hemorrhage, influenced by embolization site, material choice, and unrecognized anastomoses. Recognizing these risks is essential for refining embolization strategies and minimizing complications.

A limitation of this study is its reliance on a single case, restricting generalizability. While this is the first report of ischemic complications from an MMA-PMA anastomosis after embolization, asymptomatic cases may be underreported, underestimating the true incidence. Additionally, embolic material choice may have influenced ischemic injury. Liquid embolic agents allow real-time visualization, potentially reducing unintended distal embolization, but this was not evaluated. A concrete and evidence-based selection of embolization materials, such as a flow-chart decision, seems warranted to minimally avoid similar complications. Further research is needed to assess the frequency and impact of occult MMA-PMA anastomoses and whether different techniques or materials could mitigate associated risks.

## Conclusions

This case highlights a rare but significant complication of tumor embolization performed before meningioma resection, caused by an occult anastomosis between the MMA and the PMA. Although these anastomoses were not visible on preprocedural superselective angiography, they opened during embolization due to changes in hemodynamics. This underscores the importance of anticipating occult anastomoses in embolization planning to mitigate ischemic risks. A six-vessel study (bilateral ICA, ECA, and VA angiography) before embolization allows for a more comprehensive vascular assessment. Even if no anastomoses are identified, embolization strategies should be designed with the assumption that occult anastomoses may exist and become functionally significant during the procedure. Recognizing these potential risks and refining embolization techniques are essential to improving patient safety in meningioma treatment.
